# Unraveling of Enigmatic Hearing-Impaired *GJB2* Single Heterozygotes by Massive Parallel Sequencing: DFNB1 or Not?

**DOI:** 10.1097/MD.0000000000003029

**Published:** 2016-04-08

**Authors:** So Young Kim, Ah Reum Kim, Nayoung K. D. Kim, Chung Lee, Min Young Kim, Eun-Hee Jeon, Woong-Yang Park, Byung Yoon Choi

**Affiliations:** From the Department of Otorhinolaryngology-Head and Neck Surgery, CHA medical center, CHA university, Seongnam (SYK), Department of Otorhinolaryngology-Head and Neck Surgery (SYK, ARK), Seoul National University Hospital, Seoul National University College of Medicine; Samsung Genome Institue (NKDK, CL), Samsung Medical Center, Seoul, Korea; Department of Health Sciences and Technology (CL), SAIHST, Sungkyunkwan University, Seobu-ro, Jangan-gu, Suwon, Republic of Korea; Department of Otorhinolaryngology-Head and Neck Surgery (MYK, E-HJ, BYC), Seoul National University Bundang Hospital, Seoul National University College of Medicine, Seongnam; Samsung Genome Institute (W-YP), Samsung Medical Center; Department of Molecular Cell Biology, School of Medicine (W-YP), Sungkyunkwan University; Sensory Organ Research Institute (BYC), Seoul National University Medical Research Center, Seoul, Korea; and Wide River Institute of Immunology (BYC), Seoul National University College of Medicine, Hongcheon, Republic of Korea.

## Abstract

The molecular etiology of nonsyndromic sensorineural hearing loss (SNHL) in subjects with only one detectable autosomal recessive *GJB2* mutation is unclear. Here, we report *GJB2* single heterozygotes with various final genetic diagnoses and suggest appropriate diagnostic strategies. A total of 160 subjects with SNHL without phenotypic markers were screened for *GJB2* mutations. Single-nucleotide variants or structural variations within the DFNB1 locus or in other deafness genes were examined by Sanger sequencing, breakpoint PCR, and targeted exome sequencing (TES) of 129 deafness genes. We identified 27 subjects with two mutations and 10 subjects with only one detectable mutation in *GJB2.* The detection rate of the single *GJB2* mutation among the 160 SNHL subjects in the present study (6.25%) was higher than 2.58% in normal hearing controls in Korean. The DFNB1 was clearly excluded as a molecular etiology in four (40%) subjects: other recessive deafness genes (N = 3) accounted for SNHL and the causative gene for the other non-DFNB1 subject (N = 1) was not identified. The etiology of additional two subjects was potentially explained by digenic etiology (N = 2) of *GJB2* with *MITF* and *GJB3*, respectively. The contribution of the single *GJB2* mutation in the four remaining subjects is unclear. Comprehensive diagnostic testing including TES is prerequisite for understanding *GJB2* single heterozygotes.

## Abstract 2

Supplemental Digital Content is available in the text

## INTRODUCTION

Extreme etiologic heterogeneity exists in sensorineural hearing loss (SNHL) (http://hereditaryhearingloss.org/). Therefore, molecular genetic testing methods for efficiently screening the various deafness genes are necessary for accurately diagnosing SNHL. Various techniques, such as whole exome sequencing and targeted exome sequencing (TES), have made it possible to screen candidate genes in an extremely high-throughput manner.^1^ However, making a conclusive molecular diagnosis still requires time and rigorous effort in many cases, particularly in nonsyndromic cases with rare variants. Thus, we recently suggested the importance of phenotype-driven genetic testing focusing on candidate genes according to the clinical phenotypes of the affected subjects.^2^

The predominant mutations in *gap junction protein beta 2 (GJB2)* (MIM ID: 121011) account for up to 50% of autosomal recessive nonsyndromic SNHL in some ethnicities. This significantly reduces the molecular genetic testing loads needed to reach a final genetic diagnosis through *GJB2* testing alone.^3^ Although the proportion of nonsyndromic hearing loss and deafness 1 (DFNB1) among nonsyndromic sporadic or autosomal recessive severe to profound SNHL significantly varies, it still accounts for ∼15% of such subjects in Koreans, making *GJB2* and *solute carrier family 26, member 4 (SLC26A4)* the most frequent causative genes of SNHL.^3–7^ However, it is not always straightforward to diagnose DFNB1 in subjects carrying *GJB2* mutations. We occasionally encounter uncertain cases with a monoallelic *GJB2* mutation. It is estimated that ∼6% to 20% of identified *GJB2* mutations in hearing-impaired subjects were monoallelic mutations,^8–12^ although the value was reported to be up to 50% in some European populations.^13^ It has been speculated that a substantial portion of DFNB1 subjects had only one detectable mutation in *GJB2* and that an occult large deletion within the DFNB1 locus in *trans* with the detected *GJB2* mutation may cause deafness in these subjects.^13–15^ However, the large genomic deletion within the DFNB1 locus has not been detected in Koreans to date, suggesting that interpretation of a monoallelic *GJB2* mutation should differ depending on ethnicity. Additionally, high carrier frequencies of *GJB2* make it difficult to regard these subjects simply as DFNB1 in Korea.^4^ Moreover, some researchers have reported the digenic pathogenesis of deafness involving the detected single *GJB2* mutation in combination with mutations in other deafness genes, further complicating interpretation.^16–18^

Although numerous studies have examined the clinical implications of single heterozygous mutations in *GJB2* on SNHL, there is no consensus regarding the interpretation of the results when one mutation is identified in the *GJB2* gene of the subject. Thus, we propose the use of stepwise molecular genetic approaches to clarify the contribution of the detected monoallelic *GJB2* mutation to SNHL in Koreans based on our experiences with *GJB2* single heterozygotes.

## MATERIALS AND METHODS

### Ethical considerations

This study was approved by the institutional review boards (IRBs) at Seoul National University Hospital (IRBY-H-0905-041-281) and Seoul National University Bundang Hospital (IRB-B-1007-105-402). Written informed consent was obtained from all of the participating subjects. For children, written informed consent was obtained from their parents or guardians on their behalf.

### Clinical Evaluation

A total of 470 subjects with SNHL were subjected to genetic testing for SNHL at the otolaryngology clinics of Seoul National University Hospital and Seoul National University Bundang Hospital from September 2010 to March 2015. All of the recruited subjects were of Korean ethnicity.

Clinical characteristics of the cohort included gender, date of birth, medical history, physical examination, pure tone audiometry, and imaging studies. All of the enrolled subjects underwent audiologic evaluation using pure-tone audiometry, speech audiometry, auditory brainstem response, and auditory state response. The pure-tone thresholds were recorded at 0.25, 0.5, 1, 2, 4, and 8 kHz. However, some infants could be recorded at only limited frequencies because of poor cooperation. The hearing threshold was calculated by averaging the thresholds of 0.5, 1, 2, and 4 kHz, and was classified as subtle (16–25 dB), mild (26–40 dB), moderate (41–70 dB), severe (71–95 dB), or profound (>95 dB).

The presence of any phenotypic markers indicating syndromic SNHL was thoroughly investigated in subjects and their family members. The phenotypic markers included ophthalmologic abnormality, such as dystopia canthorum (lateral displacement of inner canthi) in Waardernburg syndrome,^19^ depigmented skin lesion, freckled face, or hypopigmentation with respect to hair color. Imaging evaluations were conducted using temporal bone computed tomography or magnetic resonance imaging to identify inner ear anomalies such as an enlarged vestibular aqueduct in subjects. All family members were also investigated for the presence of any type of hearing loss.

Based on the results of the retrieved clinical and audiologic evaluations, 160 bilateral SNHL subjects without audiologic or radiological phenotypic markers and segregating either into a sporadic or possibly autosomal recessive fashion were screened for the presence of *GJB2* mutations.

### DNA Samples and Sanger Sequencing of *GJB2* Coding Region

**FIGURE 1:**
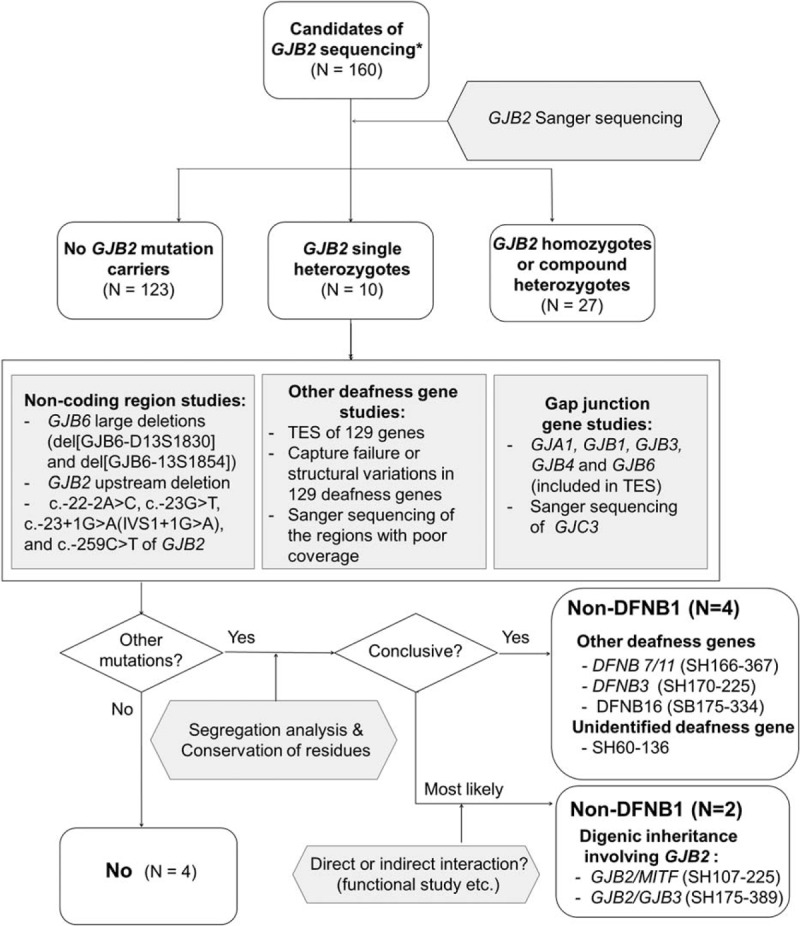
Molecular diagnostic flow of the present study: *GJB2* single heterozygotes were analyzed using a stepwise and comprehensive molecular diagnostic protocol including massive parallel sequencing of 129 known deafness genes and Sanger sequencing of additional gap junction genes as well as screening of known *GJB6* large deletions. Approximately half of *GJB2* single heterozygotes were not DFNB1. Completely different causative genes or even digenic etiology involving the *GJB2* mutation accounted for these non-DFNB1 subjects. DFNB1 = nonsyndromic hearing loss and deafness 1, GJB2 = gap junction protein beta 2, GJB6 = gap junction protein beta 6.

Genomic DNA was extracted from peripheral blood using standard protocols (Gentra Puregene Blood Kit, Qiagen, Venlo, Limburg, the Netherlands). Sanger sequencing of *GJB2* was performed for subjects without noticeable phenotypic markers as described previously.^4,6^ Further genetic tests were conducted to determine the definitive genetic etiology of SNHL in *GJB2* single heterozygotes and their family members as described below (Figure 1).

### Examination of *GJB2* Noncoding Region

To exclude the presence of pathogenic *GJB2* mutations outside of the coding regions, Sanger sequencing for the four known pathogenic mutations, c.-22-2A>C,^20^ c.-23G>T,^21^ c.-23+1G>A (formerly referred to as IVS1+1G>A),^22^ and c.-259C>T^23^ was performed using two primer sets: GJB2-AF (5′-GGCGGGAGACAGGTGTTG-3′), GJB2-AR (5′-CCAAGGACGTGTGTTGGTC-3′) and G259F (5′-AGCGCTCATAAATGCCAAGT-3′), G259R (5′-GCCGCAACACCTGTCTCC-3′).

**FIGURE 2:**
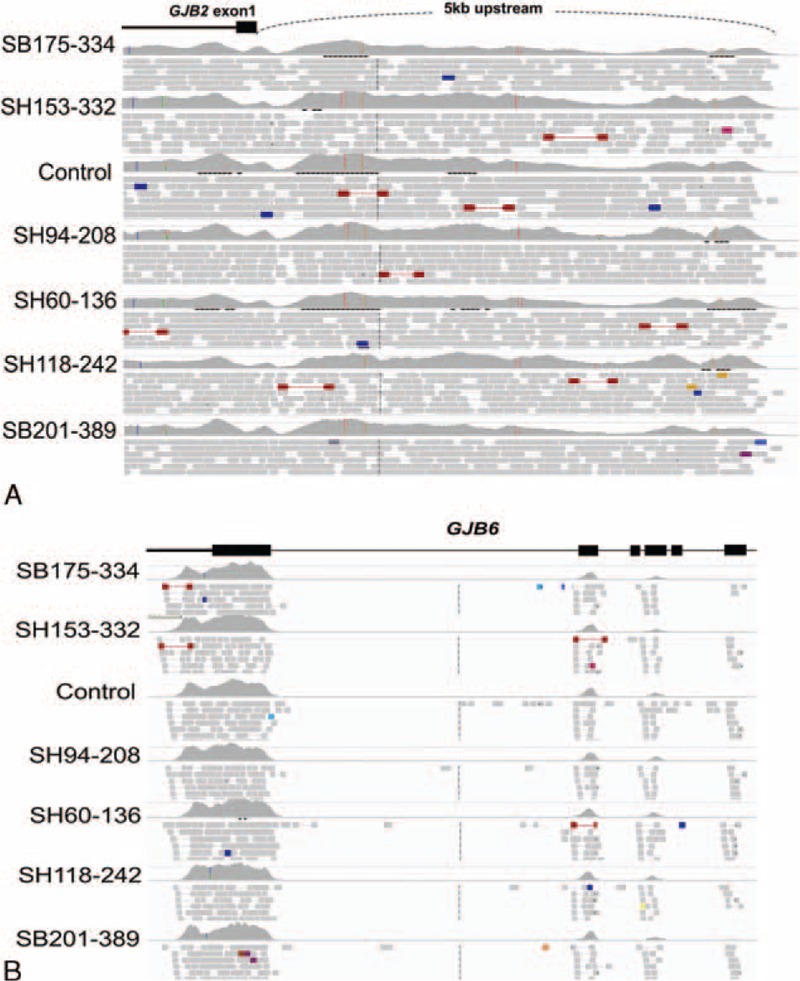
Integrative Genomic Viewer findings of noncoding upstream regions of *GJB2* (A) and *GJB6* regions (B) from six *GJB2* single heterozygotes (five group III subjects and SH60-136) with prelingual SNHL and a control with normal hearing: six *GJB2* single heterozygotes with SNHL showed no noticeable difference in terms of coverage in these regions compared with the control, indicating that no structural variations were *in trans* with the detected single heterozygous *GJB2* mutation in these regions. GJB2 = gap junction protein beta 2, GJB6 = gap junction protein beta 6, SNHL = sensorineural hearing loss.

Next, we conducted a multiplex breakpoint PCR assay for two previously reported large genomic deletions (del[GJB6-D13S1830] and del[GJB6-D13S1854]).^13^ To detect other structural variations involving 5-kb regions upstream of *GJB2* and the *GJB6* region within the DFNB1 locus, we also verified the raw data of TES using Integrative Genomic Viewer (http://www.broadinstitute.org/igv/home (Figure 2).

### Studies of Other Deafness Genes: TES and gap Junction Gene Studies

The presence of causative mutations in other deafness genes was investigated by targeted capture of exons and flanking sequences of 129 deafness genes, followed by massively parallel sequencing of DNA libraries as previously described.^24^ Variant detection was accomplished as described.^24^

To exclude the possibility of capture failure or structural variations involving the exons of any of the 129 target genes, we also analyzed the coverage status of TES during the second phase of TES data analysis. Sanger sequencing of exons showing poor coverage (<10 × ) was performed with primer sets of *Otoferlin (OTOF), Stereocilin (STRC), and Otoancorin* (OTOA) (see Table S1, Supplemental Content, which illustrates primer sequences for Sanger sequencing to screen exons of *OTOF*, *STRC*, and *OTOA* showing poor coverages by TES) so that variants that may reside in these regions could be detected (see Table S2, Supplemental Content, which illustrates regions showing significantly low depth of coverage in TES: *OTOF*, *STRC*, and *OTOA*).

Several gap junction genes, including gap junction protein alpha 1 (GJA1), gap junction protein beta 1 (GJB1), GJB2, gap junction protein beta 3 (GJB3), gap junction protein beta 4 (GJB4), and gap junction protein beta 6 (GJB6), were screened either by TES or Sanger sequencing. Particularly, Sanger sequencing of Gap junction protein gamma 3 (GJC3), which was not included in our TES deafness panel, was separately conducted to explore other mutations in gap junction genes.^25^ The GJC3 primer sequences were as follows: GJC3-1F (5′-CCTTGGATTAGGAGTGACAAGG-3′), GJC3-1R (5′-CCCTGGGACATCTGTGTTG-3′), GJC3-2F (5′-AAGGCTGCCTGCTTCGAT-3′), GJC3-2R (5′-TCTTTAGGAAAATGGTCTTCTCA-3′), GJC3-3F (5′-CCTGGGGTTGCAGTACCAC-3′), GJC3-3R (5′-TTGTACTTCCCAGAAAGGTGA-3′), GJC3-4F (5′-ATGGGTGGCACCTAAAGTGT-3′), and GJC3-4R (5′-GTCCCAGTTGTCGGTTATGC-3′).

### Investigation of Genetic Contributions of Detected Variants and/or Mutations

Segregation analysis, evaluating variants in the Korean normal hearing control group, and evaluating the conservation of residues among species were performed for variations and/or mutations obtained in the genetic studies described earlier. The possibility of digenic inheritance of SNHL resulting from the detected monoallelic *GJB2* mutation with other deafness genes was also considered based on previous studies.^16–18^

## RESULTS

### Recruitment of *GJB2* Single Heterozygotes and Clinical Evaluations

Among the 160 subjects with moderate to profound SNHL without any phenotypic markers, 37 subjects (23.1%, 37/160) harbored at least one mutant allele in *GJB2*. Twenty-seven subjects were confirmed to be DFNB1 with two definitely pathogenic mutant alleles in *GJB2* either as homozygotes or compound heterozygotes. The remaining 10 subjects (27.0%, 10/37) presented monoallelic *GJB2* mutations or suspicious variants (p.T123N from SH94-208) (Tables 1 and 2). Five kinds of *GJB2* mutations including one possibly pathogenic variant (p.T123N) were detected in these subjects: p.V37I (SB 175-334), p.R143W (SH60-136 and SB201-389), p.V193E (SH118-242, SH184-416, SH170-377, and SH175-389), c.235delC (SH153-332, SH166-387, and SH107-225), and p.T123N (SH94-208).

**TABLE 1:**
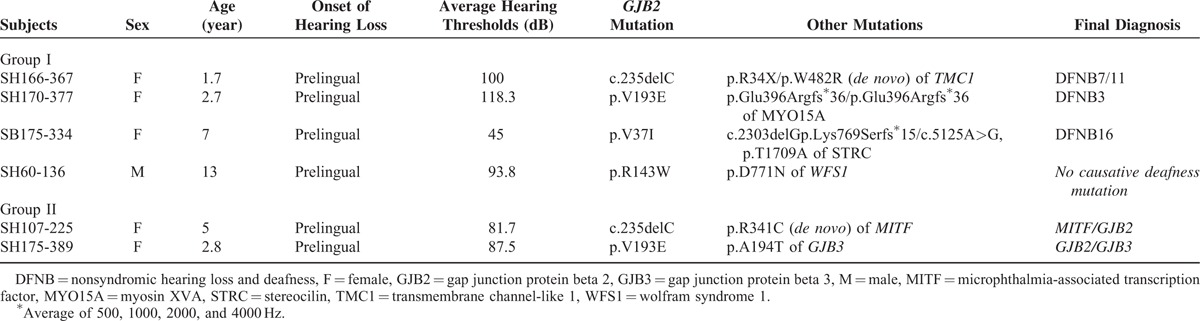
*GJB2* Single Heterozygotes where DFNB1 was Excluded as a Final Molecular Diagnosis (Groups I and II)

**TABLE 2:**
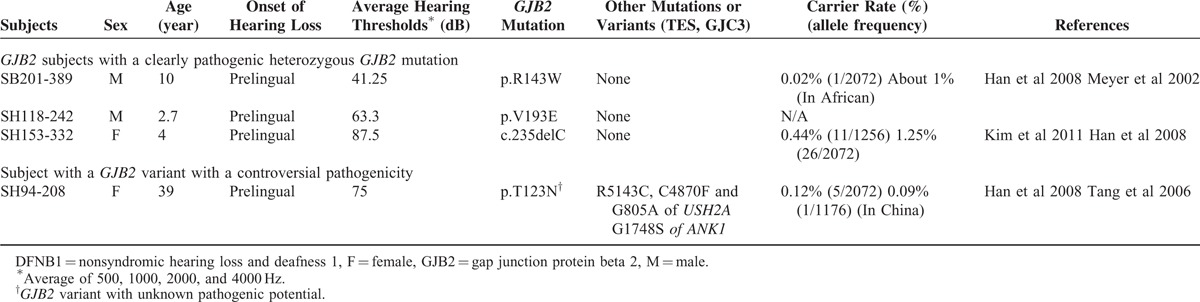
GJB2 Single Heterozygotes Where DFNB1 Could Not be Excluded (Group III)

None of the 10 single heterozygotes of *GJB2* carried pathogenic structural variations of *GJB6*/upstream regions of *GJB2* (Figure 2) or previously reported single-nucleotide variations in the noncoding regions of *GJB2* (data not shown). Next, we analyzed the TES data to identify causative point mutations of other deafness genes, if any, in these subjects.

### *GJB2* Single Heterozygotes where DFNB1 was Excluded as a Final Molecular Diagnosis: A Fortuitously Detected *GJB2* Mutation (Group I)

There were three subjects (SH166-367, SH170-377, and SB175-334) with two recessive mutations, presumed to be pathogenic, in completely different deafness genes. One of the children with a heterozygous c.235delC mutation (SH 166-367) was identified to carry a predominant founder mutation, p.R34X (c.100C>T) (rs121908073), and a novel variant, p.W482R of *Transmembrane channel-like 1 (TMC1)* (NM_138691), in a *trans* configuration (Table 1). The recessive mutation p.R34X was previously reported as a single founder mutation of *TMC1*.^27,28^ The other novel missense variant, c.1444T>C (p.W482R), was also strongly considered pathogenic because the residue was highly conserved among various species including zebrafish and *Caenorhabditis elegans* as indicated by the high GERP score (6.02). This variant was predicted to be “probably damaging” by Polyphen2 (http://genetics.bwh.harvard.edu/pph2/) based on *in silico* analyses. Furthermore, this variant was not detected among the 544 control chromosomes from normal hearing Korean subjects. Similarly, SH170-377 carrying the p.V193E mutation in *GJB2* also contained a previously reported homozygous p.Glu396Argfs^∗^36 mutant allele in *Myosin XVA (MYO15A)* (NM_016239) (Table 1).^29^

Although no other causative deafness mutation was detected in the initial analysis of TES data, Sanger sequencing for the low coverage area (<10×) in TES (see Table S2, Supplemental Content, which illustrates regions showing significantly low depth of coverage in TES: *OTOF*, *STRC*, and *OTOA*) revealed the two known pathogenic *STRC* mutations as a compound heterozygous configuration in SB175-334 (Table 1).^30^ To sum up, SH166-367, SH170-377, and SB175-334 which would have been considered DFNB1 without TES were found to be DFNB7/11, DFNB3, and DFNB16, respectively.

**FIGURE 3:**
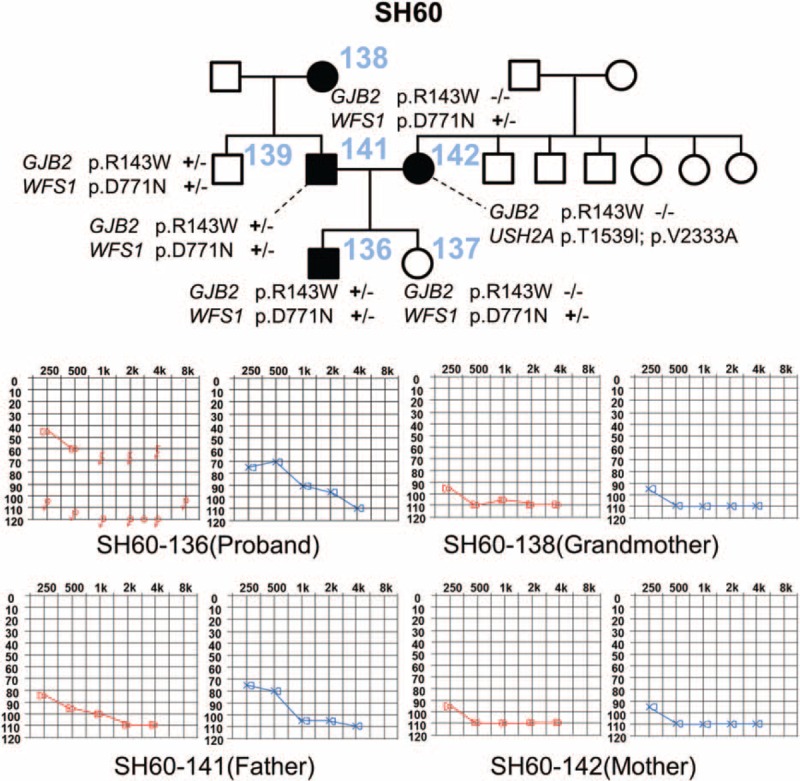
Pedigree and audiograms of SH60 and segregation of variations of *GJB2* and *WFS1* in this family: two subjects with SNHL, SH60-138 and SH60-142, showed a discrepancy in the *GJB2* genotype. Two unaffected subjects, SH60-137 and SH60-139, also carried p.D771N in *WFS1.* This indicates that neither p.R143W in *GJB2* nor p.D771N in *WFS1* contributed to SNHL in SH60-136 and that p.R143W in *GJB2* was an incidentally detected variant in this subject. GJB2 = gap junction protein beta 2, SNHL = sensorineural hearing loss, WFS1 = wolfram syndrome 1.

Finally, a subject with the heterozygous p.R143W mutation in *GJB2* (SH60-136) carried a p.D771N variant in *Wolfram syndrome 1 (WFS1)* (NM_001145853) according to TES. However, neither p.R143W in *GJB2* nor p.D771N in *WFS1* was predicted to contribute to SNHL of SH60-136 based on rigorous segregation analysis of the phenotype and the variants (Figure 3). As a result, DFNB1 was excluded for SH60-136.

### Single Heterozygous *GJB2* Mutant Allele Possibly Contributing to Deafness via Digenic Inheritance: Double Heterozygosity with Additional Mutation in Other Deafness Genes (Group II)

**FIGURE 4:**
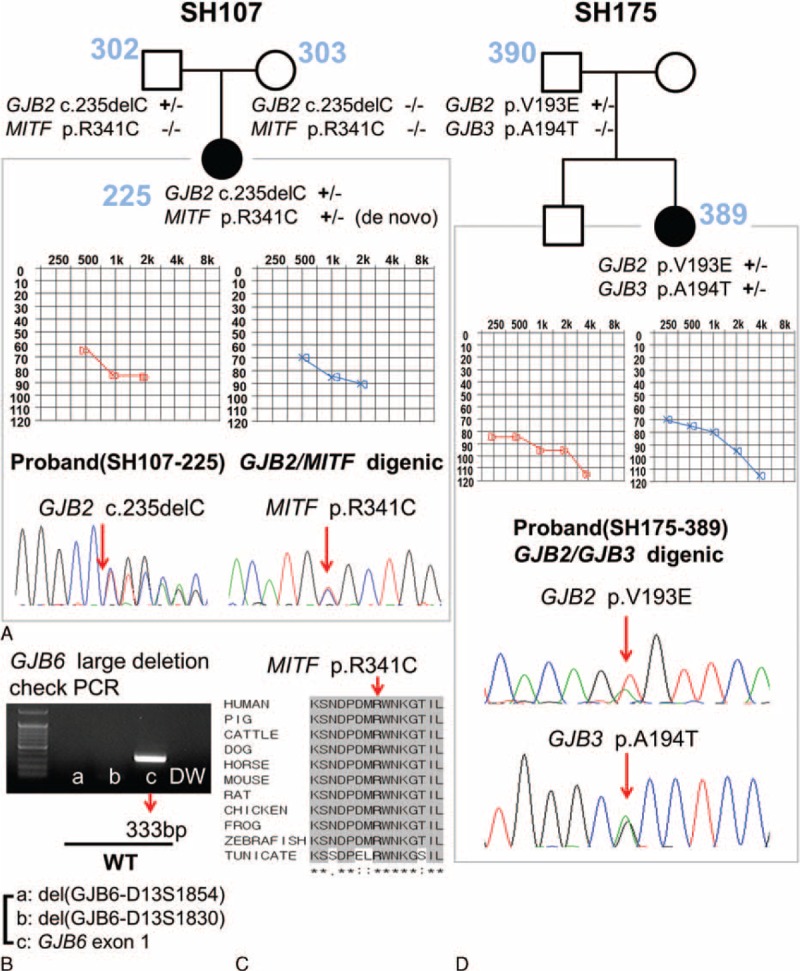
Digenic inheritances of *GJB2*/*MITF* and *GJB2*/*GJB3* (group II). (A) In addition to c.235delC in *GJB2*, the *de novo* variant of *MITF*, p.R341C was identified in SH107-225. (B) There was no *GJB6* large deletion within the DFNB1 locus. (C) The sequence of the p.R341C variant is well-conserved from humans to tunicates. (D) SH175-389 harbored a monoallelic p.V193E variant of *GJB2 and a monoallelic p.A194T variant of GJB3.* DFNB1 = nonsyndromic hearing loss and deafness 1, GJB2 = gap junction protein beta 2, GJB3 = gap junction protein beta 3, GJB6 = gap junction protein beta 6, MITF = microphthalmia-associated transcription factor.

Interestingly, two subjects (SH107-225 and SH175-389) showed double heterozygosity for a *GJB2* mutation and another likely pathogenic mutation in another deafness gene. We detected a *de novo Microphthalmia-associated transcription factor (MITF)* (NM_000248) variant, p.R341C, in one of the c.235delC carriers (SH107-225) (Figure 4A). She inherited c.235delC of *GJB2* from her father and did not have any known large genomic deletions within the DFNB1 locus (Figure 4B). The p.R341 residue of *MITF* is a well-conserved sequence among species, including zebrafish and tunicates (Figure 4C). Moreover, this *MITF* variant was not detected in the 666 control chromosomes from normal hearing Korean subjects, supporting the pathogenic potential of p.R341C in *MITF* in SH107-225. However, symptoms and signs suggesting Waardenburg syndrome type2 (WS2) including retinal abnormalities and pigmentation abnormalities could not be determined due of the patients’ young ages.

By screening other gap junction genes, another subject (SH175-389) carrying a single heterozygous p.V193E in *GJB2* allele harbored a single heterozygous p.A194T mutant allele of *GJB3* (NM_001005752) (SH175-389) with known pathogenicity (Figure 4D).^31^ This 2-year-old female showed severe autosomal recessive SNHL with a mean hearing threshold of 87.5 dB HL.

### Single Heterozygous *GJB2* Mutant Allele with Unknown Contribution to SNHL in Our Cohort (Group III)

A 39-year-old female subject (SH94-208) showed the p.T123N variant of *GJB2*. The pathogenic potential of the p.T123N variant is controversial. Three variants of *USH2A* (NM_007123)*, R5143C*, *C4870F*, and *G805A* with unknown pathogenic potential were identified using TES (see Table S3, Supplemental Content, which illustrates variants or mutations of *Usher syndrome type 2A (USH2A)* and *Ankyrin 1 (ANK1)* identified in SH 94-208). However, this subject showed no retinal abnormalities and only manifested severe SNHL with a mean hearing threshold of 75 dB HL, which was not compatible with type II Usher syndrome. Therefore, these variants of *USH2A* were excluded as causative deafness mutations. SH94-208 also showed the G1748S variant of *ANK1* (NM_000037). Structural variations such as large genomic deletions involving *ANK1* at chromosome 8p11.2p12 can lead to contiguous syndrome, with SNHL as one of the symptoms.^32^ However, the G1748S variant of *ANK1* was a point mutation, not a structural variation (see Table S4, Supplemental Content, which illustrates depth of coverage of TES). Moreover, SH94-208 showed no phenotypic markers for syndromic SNHL, such as craniometaphyseal dysplasia. Thus, this *ANK1* variant was not likely to be a causative mutation. The remaining three subjects showed no other convincingly pathogenic mutations other than the detected *GJB2* mutation (Table 2).

### Detection Rates of Single Heterozygous *GJB2* Mutation in our SNHL Cohort and a Normal Hearing Control Cohort From the Literature

Based on previously published Korean *GJB2* normal carrier rates, we compared the rates of *GJB2* single heterozygotes in the hearing-impaired cohort with that of normal hearing controls.^4,26^ Total rates (5.63% [9/160]) of *GJB2* single heterozygotes (N = 9 from group I + II + III excluding a *GJB2* variant with controversial pathogenicity [SH94-208]) among all *GJB2*-sequenced hearing-impaired subjects (N = 160) was significantly higher than that (2.58%) of normal controls (*P* = 0.03). When we excluded two cases (Group II) with the *GJB2* mutation contributing to SNHL possibly through digenic inheritance, we still observed a slightly higher rate of *GJB2* single heterozygotes (Group I + III) than in the normal controls (4.38% vs. 2.58%, *P* = 0.20), although the difference was not statistically significant.

### Analysis of Missing Regions of TES Data From *GJB2* Single Heterozygotes with Unknown Molecular Etiology

The average depth of coverage for these six subjects, including group III and SH60-136, were 225.73 (see Table S4, Supplemental Content, which illustrates depth of coverage of TES), with more than 97.09% of the target bases covered >20×. Among the 1737 regions that were targeted in this study, 2.17% were covered <20×, including several regions of *OTOF* (NM_001287489), *STRC* (NM_153700), and *OTOA* (NM_144672) (see Table S2, Supplemental Content, which illustrates regions showing significantly low depth of coverage in TES: *OTOF*, *STRC*, and *OTOA*). We confirmed that the 48th exon of *OTOF* (NM_001287489) showed very high GC content and that *STRC* and *OTOA* had pseudogenes (*STRCP1* and *LOC6537686*, respectively), which generated missing regions of the TES data. Sanger sequencing of these exons with significant poor coverage by TES revealed two compound heterozygous *STRC* mutations in SB175-334 (Table 1). However, no pathogenic variants was detected in the remaining five subjects (SH 153-332, SB201-389, SH94-208, SH60-136, and SH118-242) (data not shown).

## DISCUSSION

In the first phase analysis of TES data, we were able to exclude DFNB1 from 4 subjects (SH166-367, SH170-377, SH60-136, and SH175-389) and possibly from 1 subject (SH107-225) among a total of 10 single heterozygotes of *GJB2* in a Korean SNHL population. The second phase analysis of TES data which double-checked the exons with poor coverage in *OTOA*, *STRC*, and *OTOF* by Sanger sequencing enable us to reveal DFNB16 from one subject (SB175-334). Detection of the *GJB2* mutation in four subjects (SH166-367, SH170-377, SB175-334, and SH60-136) (Group I) was found to be incidental, independently of the phenotype. This is expected based on the similarity of the incidence rates of detection of c.235delC of *GJB2* between the one (0.63% (1 [SH166-367]/160 [our total SNHL cohort]) calculated from group I and reported figures from normal hearing Korean subjects (0.44%–1.25%) (Tables 1 and 2).^4,27^

Our first-line screening of known large genomic deletions involving *GJB6* or upstream regions of *GJB2* yielded no convincing structural variations in our population, which was in sharp contrast with previous reports of a Spanish population.^33^ In the Spanish population, 66% of the affected subjects with a monoallelic *GJB2* mutation carried the del (GJB6-D13S1830) in *GJB6*, which was also frequently observed in subjects from France and Israel.^33^ A completely different molecular etiology was clearly observed or strongly suggested in four of the five subjects in groups I and II, except SH60-136 in the present study, indicating that TES covered the known prevalent deafness genes, including gap junction genes and genes known to cause syndromic deafness such as Usher syndrome and Waardenburg syndrome for the *GJB2* single heterozygotes in Korean SNHL subjects.

For the most common *GJB2* variant in Koreans, c.235delC, suspicion of other molecular etiologies should be examined, particularly when detected as a single heterozygous state from a child with no residual hearing in Koreans, such as for SH166-367. c.235delC was recently reported to manifest a dynamic range of SNHL and a slightly milder audiologic phenotype compared with other *GJB2* variants in Koreans.^34^ Detection of mutations in *MYO15A* and *TMC1* in group I are relatively common in East Asian populations, including Koreans,^2,35–38^ indicating that application of panel sequencing covering the genes prioritized based on the ethnicity-specific prevalence would be effective for identifying *GJB2* single heterozygotes with severe to profound SNHL in Koreans.

For the family SH60 with a most likely genetic etiology but without a clear result after TES, whole exome sequencing can be used for definitive molecular diagnosis. This family SH60 segregates prelingual or perilingual severe to profound SNHL, likely in an autosomal dominant fashion, although prelingual SNHL of SH60-136 was caused by autosomal recessive mutations in other deafness genes (Figure 3). Further segregation analyses of the two variants (p.R143W and p.D771N) among the six family members of SH60 as well as clinical evaluations including audiograms excluded both p.R143W of *GJB2* and p.D771N of *WFS1* as a molecular etiology of SH60-136. Specifically, SH60-138 (grandmother of SH60-136) complained of perilingual deafness but did not carry p.R143W of *GJB2*, eliminating the contribution of this allele to prelingual profound SNHL if it segregated dominantly from SH60-138 to SH60-136 (Figure 3). Alternatively, SNHL of SH60-136 may result from autosomal recessive mutations in the same gene with SH60-142 (mother of SH60-136) if SNHL of SH60-142 was because of autosomal recessive mutations in a certain deafness gene other than *GJB2* and if the father (SH60-141) is a carrier of a mutation in the gene. In any case, p.R143W in *GJB2* does not contribute to the SNHL of SH60-136 (Figure 3). Therefore, a completely different deafness gene yet to be identified would account for the SNHL of SH60-136, warranting whole exome sequencing in this subject.

The p.V193E variant in *GJB2* occurring in complex heterozygosity with a pathogenic *GJB3* variant, p.A194T from SH175-389, suggests a possible digenic etiology of SNHL involving two different gap junction proteins, Cx26 and Cx31. Large deletions in *GJB6* (del [GJB6-D13S1830] and del [GJB6-D13S1854]) are frequently detected in a *trans* configuration with a monoallelic *GJB2* mutation in certain populations.^33,35,39^ Based on these findings, it was previously hypothesized that variations in *GJB2* and *GJB6 in trans* can cause SNHL through digenic inheritance.^33,40^ However, subsequent studies revealed that *GJB6* deletions result in an allele-specific lack of GJB2 mRNA expression, contributing to SNHL in a manner not resulting from digenic inheritance.^40^ Nevertheless, a digenic etiology involving mutations in several gap junction genes has been proposed.^18,41^ Structurally, the gap junction is composed of two connexons (Cxs), which are formed by the oligomerization of six Cx subunits. Several Cx genes, including Cx26, Cx29, Cx30, Cx31, Cx32, Cx30.3, and Cx43, can induce SNHL.^42^ These Cx gene families are known to be able to form heteromeric gap junction assemblies.^43^ In this context, digenic inheritance of SNHL involving *GJB2* (Cx26) and *GJB3* (Cx31) has been strongly supported by functional studies conducted by Liu et al (2009), which demonstrated a direct physical interaction between Cx26 and Cx31, the presence of heteromeric Cx26/Cx31 connexons, and finally co-assembly of two transfected proteins in the same junction plaques *in vitro*.^31^ In their study, two different *GJB3* mutations (p.N166S and p.A194T) were identified in three unrelated families among 108 Chinese families with a single pathogenic *GJB2* mutation.^31^ This strongly corroborated a possible digenic etiology of SNHL involving *GJB2* and *GJB3* in SH175-389. Thus, we excluded DFNB1 as a molecular etiology of SNHL from SH175-389.

Although no direct physical interaction occurs in the heterotypic Cx formation, two different genes can functionally interact with each other, which may result in functional deficit if these interactions are disrupted. *MITF* is an important gene in the development and regulation of melanocytes and is expressed in melanoblast-derived intermediate cells of the stria vascularis.^25^ Interestingly, *MITF* also regulates potassium ion circulation of endolymphatics in the inner ear.^44,45^ Although the signaling networks between *GJB2* and *MITF* remain unclear, their common final pathway in regulating potassium ion circulation in the inner ear can be significantly disrupted by the digenic effect of *MITF* and *GJB2* mutations. A subject with Waardenburg syndrome type II (WS2) in a large Chinese population had both *MITF* and *GJB2* mutations in a compound heterozygous state.^17^ The profound SNHL in the subject may have been caused by the digenic effect of *GJB2* and *MITF* mutations, although the WS2 phenotype was caused by the *MITF* mutation.^17^ In our studied family, SH107-225 with profound SNHL carried c.235delC in *GJB2* and a *de novo* variant, p.R341C in *MITF*. DFNB1 as a molecular etiology was excluded from this subject, while digenic inheritance of SNHL can be proposed for this subject because the pathogenic potential of p.R341C was strongly supported by significant conservation of the p.R341 residue among various species and by the absence of this variant among the 666 control chromosomes from normal hearing control subjects. Based on the varying degrees of audiologic phenotypes of *MITF-*related WS2, including single side deafness,^37^ bilateral profound SNHL of SH107-225 may have resulted from the additive effect of single heterozygous c.235delC of *GJB2*, and the *MITF* mutation through digenic inheritance. Thus, panel sequencing should be conducted to examine syndromic deafness genes as well as gap junction genes for definitive genetic diagnosis of *GJB2* single heterozygotes in seemingly nonsyndromic SNHL in Koreans. However, final documentation of digenic inheritance of deafness in our two subjects (SH175-389 and SH107-225) warrants a rigorous functional study.

Several exons in *OTOF*, *STRC*, and *OTOA* were not fully covered (see Table S2, Supplemental Content, which illustrates regions showing significantly low depth of coverage in TES: *OTOF, STRC, and OTOA*) by our TES data. We tried to minimize the possibility that pathogenic mutations reside in these regions by performing Sanger sequencing of all exons showing poor coverage (<10 × coverage). Considering that *STRC*-related SNHL (DFNB16) is generally presented as mild to moderate SNHL,^30^ two moderate SNHL subjects (SB175-334 and SB201-389) in our cohort were initially hypothesized to have either occult structural variations or point mutations in the poorly covered regions of *STRC*. In accordance with our assumption, one moderate SNHL subject (SB175-334) with a single heterozygous p.V37I turned out to carry two mutant alleles of *STRC*. However, it is still possible that the remaining moderate SNHL subject (SB201-389) has pathogenic structural variations in the *STRC* gene. Copy number variations of the *STRC* gene were reported to be common among deafness genes.^46^ Designing probes with different or multiple tiling on the probes may improve the capture efficiency; arrayCGH may be another option if a subject is strongly suspected to have STRC-related structural variations.

The contribution of the detected monoallelic *GJB2* mutations in four subjects in group III is unclear in the present study. The rate of *GJB2* single heterozygotes in our hearing-impaired cohort was significantly higher than that in normal hearing controls, suggesting that a substantial portion of the detected monoallelic *GJB2* mutations contributes to SNHL. Moreover, the detected *GJB2* mutation from five subjects in group I were not found to contribute to their SNHL, and thus the slightly higher rate of *GJB2* single heterozygotes in groups I + III compared with that of controls (4.38% vs 2.58%) suggests that at least some of the monoallelic *GJB2* mutations in group III contribute to SNHL.

Genetic approaches such as targeted resequencing of whole noncoding regions of *GJB2* in group III may reveal occult mutations in these regions. RNA-seq or microarray mainly focusing on gap junction genes using patient-derived lymphoblastoid cell lines may detect low or absent expression of gap junction genes either because of novel occult large deletions in these genes or disruption of regulatory elements by occult mutations in some *GJB2* single heterozygotes in group III.

At least 40% of *GJB2* single heterozygotes in Korean SNHL subjects appear to be nonDFNB1. Digenic etiology involving the *GJB2* mutation may increase the proportion. TES to cover prevalent autosomal recessive deafness genes should be employed to elucidate these non-DFNB1 *GJB2* single heterozygotes in Koreans. Similarly, a recent study also showed that ∼33.3% (4/12) of the 12 Han Chinese subjects with the monoallelic *GJB2* mutations were identified as the non-DFNB1; 2 patients with *MYO15A* mutations, 1 patient with *Potassium voltage-gated channel subfamily KQT member 4 (KCNQ4)* mutation, and 1 patient with *SLC26A4* mutations (under minor revision from “Medicine” by personal communications). Therefore, our results can be probably extrapolated to at least whole East Asian populations and our diagnostic approaches for the *GJB2* single heterozygotes can be applied to broader populations beyond Koreans. Because there have been only a small number of study subjects of *GJB2* single heterozygotes thus far, further studies with various ethnic groups and more number of subjects are warranted to generalize our diagnostic strategies. Our stepwise molecular genetic approaches presented in the present study serve as an example for understanding single heterozygotes of other autosomal recessive genes as well as of *GJB2*.

## Supplementary Material

Supplemental Digital Content

## References

[1] Kim NK Kim AR Park KT Whole-exome sequencing reveals diverse modes of inheritance in sporadic mild to moderate sensorineural hearing loss in a pediatric population. *Genet Med* 2015; 17:901–911. 25719458 10.1038/gim.2014.213

[2] Park JH Kim NK Kim AR Exploration of molecular genetic etiology for Korean cochlear implantees with severe to profound hearing loss and its implication. *Orphanet J Rare Dis* 2014; 9:167. 25373420 10.1186/s13023-014-0167-8 PMC4243193

[3] Petersen MB Willems PJ Non-syndromic, autosomal-recessive deafness. *Clin Genet* 2006; 69:371–392. 16650073 10.1111/j.1399-0004.2006.00613.x

[4] Han SH Park HJ Kang EJ Carrier frequency of GJB2 (connexin-26) mutations causing inherited deafness in the Korean population. *Human Genet* 2008; 53:1022–1028. 10.1007/s10038-008-0342-7 19043807

[5] Shin JW Lee SC Lee HK Genetic Screening of GJB2 and SLC26A4 in Korean cochlear implantees: experience of soree ear clinic. *Clin Exp Otorhinol* 2012; 5 Suppl 1:S10–S13. 10.3342/ceo.2012.5.S1.S10 PMC3369975 22701767

[6] Kim SY Park G Han KH Prevalence of p.V37I variant of GJB2 in mild or moderate hearing loss in a pediatric population and the interpretation of its pathogenicity. *PloS One* 2013; 8:e61592. 23637863 10.1371/journal.pone.0061592 PMC3636207

[7] Sagong B Baek JI Oh SK A rapid method for simultaneous screening of multi-gene mutations associated with hearing loss in the Korean population. *PloS One* 2013; 8:e57237. 23469187 10.1371/journal.pone.0057237 PMC3585873

[8] Hutchin T Coy NN Conlon H Assessment of the genetic causes of recessive childhood non-syndromic deafness in the UK—implications for genetic testing. *Clin Genet* 2005; 68:506–512. 16283880 10.1111/j.1399-0004.2005.00539.x

[9] Dai P Yu F Han B GJB2 mutation spectrum in 2,063 Chinese patients with nonsyndromic hearing impairment. *J Transl Med* 2009; 7:26. 19366456 10.1186/1479-5876-7-26 PMC2679712

[10] Yuan Y Yu F Wang G Prevalence of the GJB2 IVS1+1G >A mutation in Chinese hearing loss patients with monoallelic pathogenic mutation in the coding region of GJB2. *J Transl Med* 2010; 8:127. 21122151 10.1186/1479-5876-8-127 PMC3014891

[11] Danilenko N Merkulava E Siniauskaya M Spectrum of genetic changes in patients with non-syndromic hearing impairment and extremely high carrier frequency of 35delG GJB2 mutation in Belarus. *PloS One* 2012; 7:e36354. 22567152 10.1371/journal.pone.0036354 PMC3342211

[12] Yao J Lu Y Wei Q A systematic review and meta-analysis of 235delC mutation of GJB2 gene. *J Transl Med* 2012; 10:136. 22747691 10.1186/1479-5876-10-136 PMC3443034

[13] del Castillo FJ Rodriguez-Ballesteros M Alvarez A A novel deletion involving the connexin-30 gene, del (GJB6-d13s1854), found in trans with mutations in the GJB2 gene (connexin-26) in subjects with DFNB1 non-syndromic hearing impairment. *J Med Genet* 2005; 42:588–594. 15994881 10.1136/jmg.2004.028324 PMC1736094

[14] Lerer I Sagi M Ben-Neriah Z A deletion mutation in GJB6 cooperating with a GJB2 mutation in trans in non-syndromic deafness: a novel founder mutation in Ashkenazi Jews. *Hum Mut* 2001; 18:460. 11668644 10.1002/humu.1222

[15] Kenneson A Van Naarden Braun K Boyle C GJB2 (connexin 26) variants and nonsyndromic sensorineural hearing loss: a HuGE review. *Genet Med* 2002; 4:258–274. 12172392 10.1097/00125817-200207000-00004

[16] Tang HY Fang P Ward PA DNA sequence analysis of GJB2, encoding connexin 26: observations from a population of hearing impaired cases and variable carrier rates, complex genotypes, and ethnic stratification of alleles among controls. *Am J Med Genet Part A* 2006; 140:2401–2415. 17041943 10.1002/ajmg.a.31525 PMC3623690

[17] Yan X Zhang T Wang Z A novel mutation in the MITF may be digenic with GJB2 mutations in a large Chinese family of Waardenburg syndrome type II. *J Genet Genomics* 2011; 38:585–591. 22196401 10.1016/j.jgg.2011.11.003

[18] Kooshavar D Tabatabaiefar MA Farrokhi E Digenic inheritance in autosomal recessive non-syndromic hearing loss cases carrying GJB2 heterozygote mutations: assessment of GJB4, GJA1, and GJC3. *Int J Pediatr Otorhinolaryngol* 2013; 77:189–193. 23141803 10.1016/j.ijporl.2012.10.015

[19] Hughes AE Newton VE Liu XZ A gene for Waardenburg syndrome type 2 maps close to the human homologue of the microphthalmia gene at chromosome 3p12-p14.1. *Nat Genet* 1994; 7:509–512. 7951321 10.1038/ng0894-509

[20] Gandia M Del Castillo FJ Rodriguez-Alvarez FJ A novel splice-site mutation in the GJB2 gene causing mild postlingual hearing impairment. *PloS one* 2013; 8:e73566. 24039984 10.1371/journal.pone.0073566 PMC3765306

[21] Mani RS Ganapathy A Jalvi R Functional consequences of novel connexin 26 mutations associated with hereditary hearing loss. *Eur J Ham Genet* 2009; 17:502–509. 10.1038/ejhg.2008.179 PMC2986212 18941476

[22] Sirmaci A Akcayoz-Duman D Tekin M The c.IVS1+1G>A mutation in the GJB2 gene is prevalent and large deletions involving the GJB6 gene are not present in the Turkish population. *J Genet* 2006; 85:213–216. 17406097 10.1007/BF02935334

[23] Matos TD Caria H Simoes-Teixeira H A novel hearing-loss-related mutation occurring in the GJB2 basal promoter. *J Med Genet* 2007; 44:721–725. 17660464 10.1136/jmg.2007.050682 PMC2752183

[24] Choi BY Park G Gim J Diagnostic application of targeted resequencing for familial nonsyndromic hearing loss. *PloS One* 2013; 8:e68692. 23990876 10.1371/journal.pone.0068692 PMC3750053

[25] Locher H de Groot JC van Iperen L Development of the stria vascularis and potassium regulation in the human fetal cochlea: insights into hereditary sensorineural hearing loss. *Dev Neurobiol* 2015; 75:1219–1240. 25663387 10.1002/dneu.22279 PMC5024031

[26] Kim SY Lee BY Lim JH Determination of the carrier frequencies of selected GJB2 mutations in the Korean population. *Int J Audiol* 2011; 50:694–698. 21815880 10.3109/14992027.2011.563247

[27] Ben Said M Hmani-Aifa M Amar I High frequency of the p.R34X mutation in the TMC1 gene associated with nonsyndromic hearing loss is due to founder effects. *Genet Test Mol Biomarkers* 2010; 14:307–311. 20373850 10.1089/gtmb.2009.0174 PMC2936956

[28] Searle C Mavrogiannis LA Bennett CP The common TMC1 mutation c.100C>T (p.Arg34X) is not a significant cause of deafness in British Asians. *Genet Test Mol Biomarkers* 2012; 16:453–455. 22288896 10.1089/gtmb.2011.0254

[29] Bashir R Fatima A Naz S Prioritized sequencing of the second exon of MYO15A reveals a new mutation segregating in a Pakistani family with moderate to severe hearing loss. *Eur J Med Genet* 2012; 55:99–102. 22245518 10.1016/j.ejmg.2011.12.003 PMC3534775

[30] Vona B Hofrichter MA Neuner C DFNB16 is a frequent cause of congenital hearing impairment: implementation of STRC mutation analysis in routine diagnostics. *Clin Genet* 2015; 87:49–55. 26011646 10.1111/cge.12332 PMC4302246

[31] Liu XZ Yuan Y Yan D Digenic inheritance of non-syndromic deafness caused by mutations at the gap junction proteins Cx26 and Cx31. *Hum Genet* 2009; 125:53–62. 19050930 10.1007/s00439-008-0602-9 PMC2737700

[32] Cau M Congiu R Origa R New case of contiguous gene syndrome at chromosome 8p11.2p12. *Am J Med Genet: Part A* 2005; 136:221–222. 15948194 10.1002/ajmg.a.30814

[33] del Castillo I Villamar M Moreno-Pelayo MA A deletion involving the connexin 30 gene in nonsyndromic hearing impairment. *N Engl J Med* 2002; 346:243–249. 11807148 10.1056/NEJMoa012052

[34] Kim SY Kim AR Han KH Residual hearing in DFNB1 deafness and its clinical implication in a Korean population. *PloS One* 2015; 10:e0125416. 26061264 10.1371/journal.pone.0125416 PMC4464755

[35] Bolz H Schade G Ehmer S Phenotypic variability of non-syndromic hearing loss in patients heterozygous for both c.35delG of GJB2 and the 342-kb deletion involving GJB6. *Hear Res* 2004; 188:42–46. 14759569 10.1016/S0378-5955(03)00346-0

[36] Kalay E Karaguzel A Caylan R Four novel TMC1 (DFNB7/DFNB11) mutations in Turkish patients with congenital autosomal recessive nonsyndromic hearing loss. *Hum Mut* 2005; 26:591. 16287143 10.1002/humu.9384

[37] Yang S Dai P Liu X Genetic and phenotypic heterogeneity in Chinese patients with Waardenburg syndrome type II. *PloS One* 2013; 8:e77149. 24194866 10.1371/journal.pone.0077149 PMC3806753

[38] Wei Q Zhu H Qian X Targeted genomic capture and massively parallel sequencing to identify novel variants causing Chinese hereditary hearing loss. *J Transl Med* 2014; 12:311. 25388789 10.1186/s12967-014-0311-1 PMC4234825

[39] Esteves MC de Lima Isaac M Francisco AM Analysis of the presence of the GJB6 mutations in patients heterozygous for GJB2 mutation in Brazil. *Eur Arch Otorhinolaryngol* 2014; 271:695–699. 23553242 10.1007/s00405-013-2468-2

[40] Pallares-Ruiz N Blanchet P Mondain M A large deletion including most of GJB6 in recessive non syndromic deafness: a digenic effect? *Eur J Hum Genet* 2002; 10:72–76. 11896458 10.1038/sj.ejhg.5200762

[41] Rodriguez-Paris J Tamayo ML Gelvez N Allele-specific impairment of GJB2 expression by GJB6 deletion del (GJB6-D13S1854). *PloS One* 2011; 6:e21665. 21738759 10.1371/journal.pone.0021665 PMC3126855

[42] Rabionet R Gasparini P Estivill X Molecular genetics of hearing impairment due to mutations in gap junction genes encoding beta connexins. *Hum Mut* 2000; 16:190–202. 10980526 10.1002/1098-1004(200009)16:3<190::AID-HUMU2>3.0.CO;2-I

[43] Ahmad S Chen S Sun J Connexins 26 and 30 are co-assembled to form gap junctions in the cochlea of mice. *Biochem Biophys Res Commun* 2003; 307:362–368. 12859965 10.1016/s0006-291x(03)01166-5

[44] Price ER Fisher DE Sensorineural deafness and pigmentation genes: melanocytes and the Mitf transcriptional network. *Neuron* 2001; 30:15–18. 11343641 10.1016/s0896-6273(01)00259-8

[45] Tachibana M Cochlear melanocytes and MITF signaling. *J Investig Dermatol Symp Pro* 2001; 6:95–98. 10.1046/j.0022-202x.2001.00017.x 11764294

[46] Shearer AE Kolbe DL Azaiez H Copy number variants are a common cause of non-syndromic hearing loss. *Genome Med* 2014; 6:37. 24963352 10.1186/gm554 PMC4067994

