# Supplementary material for: Unraveling of Enigmatic Hearing-Impaired GJB2 Single Heterozygotes by Massive Parallel Sequencing: DFNB1 or Not?
Source: Medicine (Baltimore). 2016 Apr 8;95(14):e3029. doi: 10.1097/MD.0000000000003029 (PMC4998745; doi:10.1097/MD.0000000000003029)
Supplement: Supplemental Digital Content [file medi-95-e3029-s001.doc]

**Supplemental Content Table S1.** Primer sequences for Sanger sequencing to screen exons of *OTOF*, *STRC*, and *OTOA* showing poor coverages by TES.

| Gene | exon | Primer sequence (5’ - 3’) | |
| --- | --- | --- | --- |
|  |  | Forward | Reverse |
| *OTOF*  (NM_001287489) | 48 | TGTAAGCAGCAAGTCGGGTA | CCAAGGCATGAAGAGTGGAC |
| *STRC*  (NM_153700) | 1 | ATTCAGCGGCTCAGGTAACA | TTGGCCTGCTTTTCTTAACC |
|  | 3 | CCCTCTATGCTGCCTTTCAG | GACAGTCACACCCCAAAACC |
|  | 4 | GGGACCTCGCTTGTAGAAAAG | CTGTGGAGTGGCAGGCTT |
|  |  | CACCACCCTACAGCATTTCC | GGTCCAATGTGGCATGGT |
|  |  | CCTCACCCCAGACATCTTTT | CAAGAGCTGGGACAGAGGAC |
|  |  | CCATGTATGAGGTCCTGGTG | GAAAGGCATCCCAGAAGGAG |
|  |  | CTTGGCATGCTATCCCAGTT | TGCTTCAACTACTACCCCACA |
|  | 6 | CCTGCAGATTCTAGTGCAGGT | GAATGGATTCTGCCTCTGGA |
|  | 7 | CAGAGGGCTTGCTTTAGTGG | TGCTACGATGTTGGCACATT |
|  | 8 | CAGGGCTACAGAGGGTCAGA | GGATCTGAGATGGGGAAGGT |
|  | 9 | TGGGGACAAAGAGTCAATGG | GGCCACTGCCTACTCTCTTG |
|  | 10 | TGCCCCTGTGTAGTGGTGTA | AATGGAGGTTGCAAAAGTGG |
|  | 11 | TCCAATCCCTAGTTCCAAACC | CGTCCTCCTGCAGACAGAGT |
|  | 15 | ACCCCCATGGTTCCTTTTCT | CTGGCCAAATAGGGCTAAGG |
|  | 29 | AGGGAGATCCCTGGAGTG | CAGTGGGATTATTTATGATGGAAA |
| *OTOA*  (NM_144672) | 22 | TGACTGGCAAAGAATGTTACTCA | GATGATGTATGGGAAAACTGCTC |
|  | 23 | AGGCCCAACCTTCCAATACT | AAACAGACTCAAAAATGACCATCA |
|  | 24 | GGATGGGAGATCAGTCAGGA | AAGGTGCCAAGTGTCAGGAG |

**Supplemental Content Table S2 Regions showing significantly low depth of coverage in TES: *OTOF*, *STRC*, and *OTOA*.**

| Gene | exon | Location | SH153-332 | SB201-389 | SB175-334 | SH94-208 | SH60-136 | SH118-242 |  | Average | Mapping Quality |
| --- | --- | --- | --- | --- | --- | --- | --- | --- | --- | --- | --- |
|  |  |  | depth (×) | | | | | | | |  |
| *OTOF*  (NM_001287489) | 48 | chr2:26680908-26681088 | 12.81 | 10.96 | 9.65 | 16.28 | 6.70 | 11.89 |  | 11.38 | 60 |
| *STRC*  (NM_153700) | 1 | chr15:43910839-43910938 | 0 | 0 | 0 | 0 | 0 | 0 |  | 0 | 0 |
|  | 3 | chr15:43909619-43909718 | 0.31 | 0 | 1.70 | 1.30 | 1.57 | 0 |  | 0.81 | 0 |
|  | 4 | chr15:43907631-43908888 | 0.08 | 0.09 | 0.48 | 0.46 | 0.69 | 0.14 |  | 0.32 | 0 |
|  | 6 | chr15:43906394-43906510 | 2.54 | 8.47 | 7.75 | 13.75 | 17.88 | 13.36 |  | 10.63 | 0 |
|  | 7 | chr15:43906085-43906251 | 0.43 | 0.40 | 0.78 | 1.19 | 0.10 | 2.56 |  | 0.91 | 0 |
|  | 8 | chr15:43905243-43905429 | 2.01 | 0.45 | 6.18 | 11.45 | 11.16 | 0.04 |  | 5.21 | 0 |
|  | 9 | chr15:43904989-43905104 | 0 | 0 | 0 | 0 | 0 | 0.87 |  | 0.15 | 0 |
|  | 10 | chr15:43904575-43904707 | 0 | 0 | 0 | 0 | 0 | 0 |  | 0 | 0 |
|  | 11 | chr15:43904040-43904222 | 1.98 | 0 | 4.37 | 13.80 | 7.98 | 0 |  | 4.69 | 0 |
|  | 15 | chr15:43902510-43892635 | 3.32 | 6.67 | 6.08 | 5.01 | 4.67 | 5.08 |  | 5.14 | 0 |
|  | 29 | chr15:43891864-43891964 | 3.10 | 2.17 | 9.58 | 10.77 | 8.54 | 5.30 |  | 6.58 | 0 |
| *OTOA*  (NM_144672) | 22 | chr16:21752042-21752229 | 0 | 0 | 0 | 0 | 0 | 0 |  | 0 | 0 |
|  | 23 | chr16:21756202-21756357 | 0 | 0 | 0 | 0 | 0 | 0 |  | 0 | 0 |
|  | 24 | chr16:21653256-21763398 | 9.97 | 2.65 | 2.78 | 7.19 | 16.09 | 8.43 |  | 7.85 | 0 |

**Supplemental Content Table S3** Variants or mutations of *USH2A* and *ANK1* identified in SH 94–208.

| **Ref ID** | **NC_000001** | **NC_000001** | **NC_000001** | **NC_000008** |
| --- | --- | --- | --- | --- |
| **Ref Pos** | 215802248 | 215821046 | 216420322 | 41526060 |
| **Gene Name** | USH2A | USH2A | USH2A | ANK1 |
| **Ref Seq** | G | C | C | C |
| **dbSNP ID** | 145771342 |  |  |  |
| **GERP Score** | 3.8 | 5.8 | 5.65 | 3.33 |
| **Genotype** | Hetero. Ref. | Hetero. Ref. | Hetero. Ref. | Hetero. Ref. |
| **Nucleotide Change** | G15427A | C14609A | G2414C | G5242A |
| **Protein Change** | R5143C | C4870F | G805A | G1748S |
| **SIFT** | Damaging | Damaging | Damaging | Tolerated |
| **Polyphen2** | Benign | Probably Damaging | Probably Damaging | Probably Damaging |
| **Control Study (alleles)** | 0/384 | 0/384 | 2/384 | 0/384 |
| **Depth** | 98 | 6 | 59 | 63 |
| **P not ref** | 100.00% | 92.79% | 100.00% | 100.00% |
| **Q call** | 60 | 11.098 | 60 | 60 |
| **Reported** | Huang et al., 2013 Plos One | Novel | Novel | Novel |

**Supplemental Content Table S4** Depth of coverage of TES

**.**

| Total number of total targeted regions (no.) | 1737 | | | | | |
| --- | --- | --- | --- | --- | --- | --- |
| Subject | SH153-332 | SB201-389 | SB175-334 | SH94-208 | SH60-136 | SH118-242 |
| mean depth (×) | 167.4633 | 193.7471 | 223.4573 | 317.1662 | 298.9466 | 153.5942 |
| # of region < 20× (no.) | 55 | 59 | 40 | 20 | 18 | 34 |
| # of region < 20× (percentage) | 3.17% | 3.40% | 2.30% | 1.15% | 1.04% | 1.96% |
